# Impact of a multifaceted program to prevent postoperative delirium in the elderly: the CONFUCIUS stepped wedge protocol

**DOI:** 10.1186/1471-2318-11-25

**Published:** 2011-05-18

**Authors:** Christelle Mouchoux, Pascal Rippert, Antoine Duclos, Thomas Fassier, Marc Bonnefoy, Brigitte Comte, Damien Heitz, Cyrille Colin, Pierre Krolak-Salmon

**Affiliations:** 1 Pharmacie, Hôpital des Charpennes, Hospices Civils de Lyon, Villeurbanne, France; 2 Pôle Information Médicale Évaluation Recherche, Hospices Civils de Lyon, Lyon, France; 3 EA Santé-Individu-Société 4129, Université de Lyon, Lyon, France; 4 Service de Médecin gériatrique, Equipe Mobile de Gériatrie, Centre hospitalier Lyon-Sud, Hospices Civils de Lyon, Pierre-Bénite, France; 5 Unité CarMeN, Université Claude Bernard Lyon 1, Villeurbanne, France; 6 Service de Médecine gériatrique, Equipe Mobile de Gériatrie, Hôpital Edouard Herriot, Hospices Civils de Lyon, Lyon, France; 7 Pôle de Gériatrie, Equipe Mobile de Gériatrie, Hôpitaux Universitaires de Strasbourg, Strasbourg, France; 8 Hôpital des Charpennes, Hospices Civils de Lyon, Villeurbanne, France; 9 Université Claude Bernard Lyon 1, Villeurbanne, France; 10 Inserm U821, Centre hospitalier Le Vinatier, Bron, France

## Abstract

**Background:**

Postoperative delirium is common in the elderly and is associated with a significant increase in mortality, complications, length of hospital stay and admission in long care facility. Although several interventions have proved their effectiveness to prevent it, the Cochrane advises an assessment of multifaceted intervention using rigorous methodology based on randomized study design. Our purpose is to present the methodology and expected results of the CONFUCIUS trial, which aims to measure the impact of a multifaceted program on the prevention of postoperative delirium in elderly.

**Method/Design:**

Study design is a stepped wedge cluster randomized trial within 3 surgical wards of three French university hospitals. All patients aged 75 and older, and admitted for scheduled surgery will be included. The multifaceted program will be conducted by mobile geriatric team, including geriatric preoperative consultation, training of the surgical staff and implementation of the *Hospital Elder Life Program*, and morbidity and mortality conference related to delirium cases. The primary outcome is based on postoperative delirium rate within 7 days after surgery. This program is planned to be implemented along four successive time periods within all the surgical wards. Each one will be affected successively to the control arm and to the intervention arm of the trial and the order of program introduction within each surgical ward will be randomly assigned. Based on a 20% reduction of postoperative delirium rate (ICC = 0.25, α = 0.05, β = 0.1), three hundred sixty patients will be included i.e. thirty patients per service and per time period. Endpoints comparison between intervention and control arms of the trial will be performed by considering the cluster and time effects.

**Discussion:**

Better prevention of delirium is expected from the multifaceted program, including a decrease of postoperative delirium, and its consequences (mortality, morbidity, postoperative complications and length of hospital stay) among elderly patients. This study should allow better diagnosis of delirium and strengthen the collaboration between surgical and mobile geriatric teams. Should the program have a substantial impact on the prevention of postoperative delirium in elderly, it could be extended to other facilities.

**Trial registration:**

ClinicalTrials.gov: NCT01316965

## Background

Postoperative delirium is a common acute disorder of attention and cognition, which can cause serious health concern among the elderly [1]. After hip-fracture repair, its incidence ranges from 26 to 52% [2-4]. Inpatient's delirium is associated with an increase in postoperative complications and mortality, risk of functional decline, dementia, length of hospital stay, hospital costs and rates of nursing home placement on discharge [5-11].

The impact of prevention program of delirium has been assessed in general medicine or in surgical ward. However published studies in surgical wards were focused on orthopaedic care and assessed mainly pharmacological interventions [12-14]. Yet, pharmacological interventions did not show evidence to prevent delirium in elderly [13]. Conversely, there is now increasing evidence that delirium can be prevented through non-pharmacological interventions [15]. Various educational and non-educational approaches aiming at preventing postoperative delirium have been implemented and tested in the hospital setting. A controlled study in general-medicine wards showed the effectiveness of the *Hospital Elder Life Program *(HELP) to reduce the rate of delirium by one third in elderly patients [16]. This program had been successfully reproduced in other settings [17,18]. Another randomized study proved the substantial impact of a proactive geriatric consultation preoperatively or within 24 hours postoperatively on severe delirium incidence following hip fracture surgery repair [19]. Although the implementation of a multifaceted intervention would be more effective in preventing delirium than program based on a single component, the value of complex interventions has still not been rigorously assessed [13,14]. The Cochrane advises an assessment of multifaceted intervention using rigorous methodology based on randomized study design [12].

Geriatric pathways have been reinforced since 2002 in France through creation of Mobile Geriatric Teams (MGT) in acute care [20]. The MGT expertise is essential to guaranty appropriate care and improve the management of elderly inpatients. The MGT missions include: a) patient clinical, psychological and social assessments, b) advice, information and training of health care professionals, c) implementation of care and life projects, d) management within the intra-hospital pathways and at the hospital discharge, and e) organization of the outpatient [21,22].

The purpose of this paper is to provide the detailed methodology of the CONFUCIUS stepped wedge trial. The CONFUCIUS trial aims to assess a delirium multifaceted prevention program implemented and coordinated by a MGT. Main objective is to measure the impact of a multifaceted prevention program on the incidence of postoperative delirium in elderly patients. Secondary objectives aim to assess the impact of the program on delirium intensity, postoperative complications, length of hospital stay, and exploring the program feasibility.

## Methods/Design

### Study design

**Figure 1:**
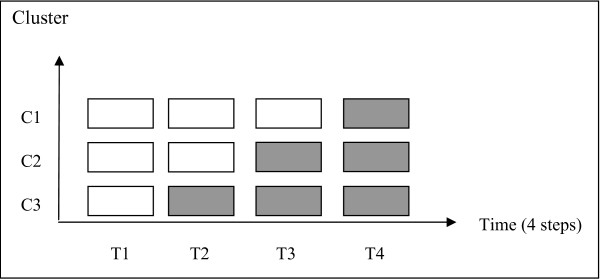
**Design of the CONFUCIUS study**. One cluster (C1, C2 or C3) represents one surgical ward. One unit (control or intervention) represents one time period of one cluster. "White square": "control" unit = surgical wards without multifaceted prevention program "Black square": "intervention" unit = surgical wards with multifaceted prevention program

CONFUCIUS protocol is based on a stepped wedge trial design, which is appropriate in assessing the impact of patient safety interventions [23,24]. It is an original design of cluster randomised controlled trial (figure 1). In the CONFUCIUS trial, the multifaceted prevention program will be rolled-out sequentially to three surgical wards (clusters) over four time periods (steps). The order in which the different surgery wards will receive the program is determined at random, allowing the full implementation of intervention in all participating clusters by the end of the trial [24]. Stepped wedge design also has strong methodological value to reduce contamination bias, considering that each cluster acts as its own control and hence provides data in both control and intervention arms of the trial [24].

### Setting and patients

The CONFUCIUS trial will take place in 3 surgical wards (orthopaedic, digestive and urology surgery) of 3 distinct French university hospitals. Each hospital benefits from its own MGT, which is composed of geriatricians and nurses. All health professionals in the participating surgical wards will be involved to conduct the multidisciplinary prevention program (i.e. surgeons, anaesthetists, nurses, and nurse assistants).

Inclusion criteria are age 75 and older, admission for a scheduled surgery (i.e. respectively colorectal cancer, ureterostomy, nephrectomy or cystectomy, total hip or knee replacement), participation agreement and no psychiatric trouble.

### Intervention

**Figure 2:**
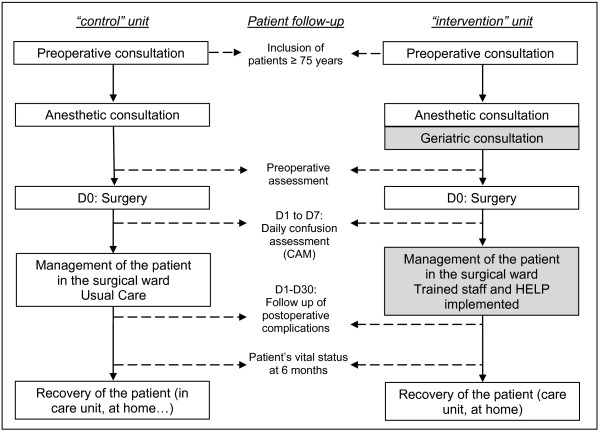
**Schema of the management of elderly patients included in the CONFUCIUS study in surgical wards during "control" and "intervention" units**. The specific patient management due to CONFUCIUS study are represented by grey boxes.

The multidisciplinary prevention program will be implemented and coordinated locally by the MGTs (table 1). Within each surgical ward, the components of this program will be introduced simultaneously (figure 2). 1/Preoperative geriatric consultation, 2/Training of the surgical wards staff and implementation of HELP in surgical wards and 3/A periodic morbidity and mortality conference related to delirium cases are detailed below.

**Table 1:** Role of the surgical wards and MGT staff during the CONFUCIUS study

Geriatrician from MGT
Training nurses from surgical wards to the CAM

Training the surgical wards staff o the prevention of postoperative delirium

Perform a preoperative geriatric consultation

Support HELP implementation

Involve a quarterly morbidity and mortality conferences

**Nurse from MGT**

Perform preoperative assessment

Perform MDAS diagnosis for CAM positive patients

**Nurse from the surgical wards**

Perform CAM from the day before the surgery until 7 days after surgery

Involve in HELP implementation

Involve in quarterly morbidity and mortality conferences

**Other medical staff from the surgical wards**

Involve in HELP implementation

Involve in periodic morbidity and mortality conferences

#### 1/Preoperative geriatric consultation

Structured geriatric consultation will be performed by geriatricians of the MGT before surgery, including: 1/medical comorbidity assessment; 2/clinical examination, 3/decrease of unnecessary and redundant medications (e.g. anticholinergics, antihistamines); 4/assessment of pain using Numeric Rating Scale (NRS) [25]; 5/assessment of nutritional status using the Mini Nutritional Assessment (MNA) [26]; 6/assessment of self-care functions using Activities of Daily Living (ADL) [27], Instrumental Activities of Daily Living (IADL) [28]and, the "Get-Up and Go" test [29]; 7/assessment of cognitive and depression status using the Mini-Mental State Examination (MMSE) [30] and the Mini Geriatric Depression Scale (Mini-GDS) [31]; 8/delirium assessment using the Confusion Assessment Method (CAM) diagnostic algorithm [32,33] and delirium severity using the Memorial Delirium Assessment Scale (MDAS) [34]. According to the geriatric consultation results and to prevent postoperative confusion, geriatricians will formulate advices on the patient care management during the peroperative period.

#### 2/Training of the surgical wards staff and implementation of the Hospital Elder Life Program (HELP)

All members of medical and nursing staffs will attend a two hours training session performed by the MGTs in their surgical ward. This training will focus on post-operative delirium issues (diagnosis, frequency, seriousness, and risk factors) and the HELP presentation [16]. This non pharmacological program focuses on patient spatiotemporal orientation, including: 1/to promote the use of appropriate glasses and hearing aids; 2/to encourage to bring personal belongings; 3/to encourage proxies to visit the patient during his hospital stay; 4/to set up clocks and calendars in patients room; 5/to adjust the luminosity according on the time of the day; 6/to improve patient implantation in the current reality (for example, telling about the time and the date during medical and nursing cares).

All these recommendations will be reminded by posters on the wall of patient rooms in surgical wards and explained by brochures provided to proxies.

#### 3/Morbidity and mortality conferences related to delirium cases

Morbidity and mortality conference will be organized in surgical ward. On a quarterly basis, they will gather all members of medical and nursing staffs and MGTs to analyze medical records of patients having experienced a postoperative delirium. Their aims are to identify and address the prevention program failures.

### Comparator

Prevention of the postoperative delirium on the control group will be according to usual care of surgical wards (figure 2). Surgical wards staffs will be able to contact the MGT spontaneously, without implementation of any systematic preoperative geriatric consultation, staff training, HELP implementation or morbidity and mortality conference.

### Outcome measurement

The primary outcome of the CONFUCIUS trial is based on postoperative delirium rate within 7 days after surgery. Secondary outcomes are the following: mean delirium intensity, length of hospital stay, postoperative complications 30 days after surgery incidence, mortality 6 months after surgery and feasibility of the multidisciplinary prevention program.

Delirium will be assessed using the CAM, which has been validated in the elderly and is highly sensitive (94-100%) and specific (90-95%) [32,33]. The CAM includes four features: 1) acute onset and fluctuating course, 2) inattention, 3) disorganization of thinking and 4) altered level of consciousness. The diagnosis of delirium requires the presence of features 1 and 2 and either 3 or 4. Its feasibility is good. It can be completed in less than 5 minutes and is well understood by physicians, nurses and trained interviewers. It will be performed by surgical ward nurses from the first to the seventh days after surgery.

Delirium intensity will be assessed using the MDAS (scored 0-30, 30 worst) [34]. The MDAS will be completed by the MGT for all CAM positive patients. Postoperative complications (e.g. pulmonary and cardiac complications, deep vein thrombosis, sepsis, fall, incontinence or retention, pressure ulcer, patient who pull off catheter, infusion and dressing) and length of hospital stay will be collected from medical records.

Compliance of surgical team to the CONFUCIUS program will be assessed for each patient by an external audit. The completion of preoperative consultation file, the appropriate use of glasses and hearing, the presence of personal belongings, clock and calendar in patient room will be checked by a research assistant.

### Data collection

The surgeons will screen all patients for trial eligibility during preoperative surgical consultation. All patients will undergo preoperative assessment the day before surgery, including a medical interview and recorded audit by a trained research team. Components of the patient interview are resumed by: 1/sociodemographic data, 2/information regarding to tobacco use, alcohol abuse, impairment of hearing and vision, 3/medical past and comorbidities, 4/assessment of pain using the NRS [25]; 5/assessment of nutritional status using the MNA [26]; 6/assessment of self-care functions using the ADL [27] and the IADL [28]; 7/assessment of cognitive and depression status using the MMSE [30] and the Mini-GDS [31]; 8/delirium assessment using CAM [32,33]. Data about past medical, biology results, surgery (type, duration, complications during surgery) and anesthesia (type of anesthesia - general versus regional, anesthetic drug used) will be obtained from the medical file by research team.

### Sample size

Sample size calculation was inspired from the methodology of Hussey and Hughes [23] and based on the following hypothesis: 1/three surgical wards (clusters) over four successive time periods, 2/improvement of at least 20% regarding primary outcome after the program implementation (from 60% to 40%) [1,16,19], 3/alpha fixed at 5%, 4/a number of patients per time period ranging from 60 to 120, 5/a coefficient of variation or intracluster correlation coefficient (ICC) ranging from 0 to 0,4. The ICC is a measure of the relatedness of clustered data by comparing the variance within clusters with the variance between clusters. Table 2 presents the statistical power obtained according to various values for ICCs and numbers of patients per time period. At least 90 patients per time period, i.e. 30 per time period and per surgical ward will be necessary to obtain a statistical power greater than 90%. Consequently, 360 patients will be included in the study. The time period duration will be 6 months to ensure the inclusion of 30 patients per time period and per surgical ward.

**Table 2:** Statistical power calculated from intracluster correlation coefficients (ICC) and numbers of patients per time period

ICC	60 patients per time period	90 patients per time period	120 patients per time period
**0**	0.95	0.99	1

**0.1**	0.87	0.96	0.99

**0.2**	0.84	0.95	0.98

**0.3**	0.83	0.94	0.98

**0.4**	0.83	0.94	0.98

### Statistical analysis

Univariate analyses using Student's test or non-parametric tests (quantitative data) and Khi^2 ^test or Fisher's exact test (qualitative data) will be performed to describe population characteristics [35].

Outcomes in all "control" and "intervention" units will be performed using multivariate analyses, adjusting on various potential confounding factors about patient characteristics. The variability of patients within the same cluster should be inferior to the inter-cluster variability. Outcomes will be assessed at the patient level by taking into account cluster effect using logistic regression. Evolution of intervention effect will be assessed by a modelling of chronological series unit by unit during the "intervention" time periods (figure 1) [36].

Only patient with at least one preoperative and one postoperative collected CAM will be analysed. An alpha level of 0.05 will be used to determine statistical significance. Data analysis will be performed using the analysis software SAS (version 9.1, SAS Institute Inc, Cary, USA).

### Ethical consideration

The participating patients will give their verbal informed consent after being told about the study. Their participation or refusal will not affect their medical and nursing care. The study protocol has been reviewed and approved by an ethics committee (Comité de Protection des Personnes Sud-Est IV). All procedures are in accordance with the declaration of Helsinki.

CONFUCIUS study has been registered in the clinical trials (Current Controlled Trials NCT01316965).

## Discussion

The implementation of the CONFUCIUS program may decrease the occurrence and severity of postoperative delirium leading to the decrease of mortality and postoperative complications in the elderly patients. A decrease of the length of hospital stay may also be disclosed after implementation of the program. The CONFUCIUS study will develop or strengthen the collaboration between surgical wards and MGTs that has already been observed during the preparative time of the study. A better recognition and management of postoperative delirium are expected by surgical ward staffs.

The CONFUCIUS study may have several limitations. Firstly, also the emergency surgery is a risk factor of postoperative delirium, patients admitted for an emergency surgery will not be included in the study. For feasibility reasons, the preoperative geriatric consultation could not be systematically performed in emergency. According to the impact of the multidisciplinary prevention program on postoperative delirium in patients with a scheduled surgery, an adaptation of CONFUCIUS program could be considered for patients admitted via the emergency room. Secondly, the CONFUCIUS program can only be implemented in hospital with MGT and its impact could be linked to MGT performance. However, the geriatric consultation and HELP will be standardized to limit biases.

The CONFUCIUS program associates three complementary interventions focusing on several stages of the patient management: (1) a preoperative geriatric consultation, (2) training of the surgical wards staff with implementation of the HELP, (3) periodic morbidity and mortality conferences. The two first components have been proven individually efficient to prevent postoperative delirium [16,19]. Introduced first in the beginning of the XX^th ^century in USA, morbidity and mortality conference represents a pedagogical method for physicians to analyze and improve health care quality [37,38].

The CONFUCIUS study is based on an original methodology. Stepped wedge trial design is a particular cluster randomised controlled study design which is useful for evaluation of patient safety interventions [23,24]. This design differs from both parallel and cross-over designs. With stepped wedge design, intervention is introduced to participants sequentially so that, by the end of the study, all participants are exposed to the intervention [23,24]. The three main motivations for using a stepped wedge design are ethical, logistical and statistical [23,24]. (1) From an ethical point of view, if the hypothesis is that the intervention will do more good than harm, it might be considered unethical to exclude any participants from the program. (2) Stepped wedge design is also particularly useful when, for logistical reasons, it is not possible to implement an intervention to all participants simultaneously. (3) Furthermore the stepped wedge design has statistical advantages. First, surgical wards act as their own control and hence provide date points in both control and intervention units [23,24]. This feature of the stepped wedge design reduces the risk of bias, which may be most important in non-randomised studies. Second, the effects of time can be included in the statistical model, hence controlling for temporal changes in the effectiveness of the program [23,24].

## Conclusion

Impact of programs on delirium has already been assessed in general medicine or in surgical wards; however surgical ward studies were performed only in orthopaedics and assessed mainly the impact of pharmacological interventions. The CONFUCIUS study strengths are the following: it takes place in different surgical wards (orthopaedic, urology and digestive surgery), it assesses a multifaceted intervention and it uses an original stepped wedge randomised controlled trial. Should the program have a substantial impact on the prevention of postoperative delirium in elderly, it could be extended to other facilities.

## Competing interests

The authors declare that they have no competing interests.

## Authors' contributions

CM, PR, AD, TF, CC and PKS designed and managed the study. CM, PR wrote the main part of the article. The critical revision of the manuscript was made by CM, PR, AD, TF, MB, BC, DH, CC, PKS. All authors have read and approved the final manuscript. CM, AD, CC and PKS were obtained the funding.

## Pre-publication history

The pre-publication history for this paper can be accessed here:

http://www.biomedcentral.com/1471-2318/11/25/prepub
